# Host Growth Can Cause Invasive Spread of Crops by Soilborne Pathogens

**DOI:** 10.1371/journal.pone.0063003

**Published:** 2013-05-08

**Authors:** Melen Leclerc, Thierry Doré, Christopher A. Gilligan, Philippe Lucas, João A. N. Filipe

**Affiliations:** 1 Institute for Genetics Environment and Plant Protection, Institut National de la Recherche Agronomique – Agrocampus Ouest – University of Rennes 1, Le Rheu, France; 2 Agronomy group, AgroParisTech, Thiverval-Grignon, France; 3 Agronomy Group, Institut National de la Recherche Agronomique, Thiverval-Grignon, France; 4 Epidemiology and Modelling Group, Department of Plant Sciences, University of Cambridge, Cambridge, United Kingdom; University of Helsinki, Finland

## Abstract

Invasive soilborne plant pathogens cause substantial damage to crops and natural populations, but our understanding of how to prevent their epidemics or reduce their damage is limited. A key and experimentally-tested concept in the epidemiology of soilborne plant diseases is that of a *threshold spacing* between hosts below which epidemics (invasive spread) can occur. We extend this paradigm by examining how plant-root growth may alter the conditions for occurrence of soilborne pathogen epidemics in plant populations. We hypothesise that host-root growth can 1) increase the probability of pathogen transmission between neighbouring plants and, consequently, 2) decrease the threshold spacing for epidemics to occur. We predict that, in systems initially below their threshold conditions, root growth can trigger soilborne pathogen epidemics through a switch from non-invasive to invasive behaviour, while in systems above threshold conditions root growth can enhance epidemic development. As an example pathosystem, we studied the fungus *Rhizoctonia solani* on sugar beet in field experiments. To address hypothesis 1, we recorded infections within inoculum-donor and host-recipient pairs of plants with differing spacing. We translated these observations into the individual-level concept of pathozone, a host-centred form of dispersal kernel. To test hypothesis 2 and our prediction, we used the pathozone to parameterise a stochastic model of pathogen spread in a host population, contrasting scenarios of spread with and without host growth. Our results support our hypotheses and prediction. We suggest that practitioners of agriculture and arboriculture account for root system expansion in order to reduce the risk of soilborne-disease epidemics. We discuss changes in crop design, including increasing plant spacing and using crop mixtures, for boosting crop resilience to invasion and damage by soilborne pathogens. We speculate that the disease-induced root growth observed in some pathosystems could be a pathogen strategy to increase its population through host manipulation.

## Introduction

Invasions by plant pathogens can significantly impact plant communities [1], [2], [3] and cause substantial economic losses in agricultural and silvicultural systems [4], [5]. Epidemiological modelling can play an important role in designing and evaluating strategies for preventing and controlling pathogen invasions [6], [7]. For example, mathematical models allow the prediction of *threshold conditions* for pathogen invasion (epidemics) in host populations [8], [9], [10] on which some criteria for optimal disease control are based [6], [11].

For most soilborne plant diseases, pathogen spread occurs predominantly between plants that have grown as close neighbours [7], [12], [13], [14], posing stricter threshold conditions for pathogen invasion than in well mixed populations, e.g., a higher transmission rate [12], [15]. In these systems, there is a close association with the concept of *percolation threshold* [16], i.e., a critical probability of connection (and transmission, in our case) between neighbour sites in a lattice. The existence of these thresholds for soilborne disease epidemics has been shown, for example, in laboratory conditions [17]. The spatial distribution and density of host populations also determine epidemic thresholds. While, the effects of these factors on epidemics of locally-spreading plant pathogens have been investigated by several authors [12], [14], [18], [19], the corresponding effects of host growth have received little attention [20], [21]. In this paper, we investigate experimentally and theoretically, how host growth can alter epidemic thresholds (invasive spread) for soilborne plant pathogens.

**Figure 1:**
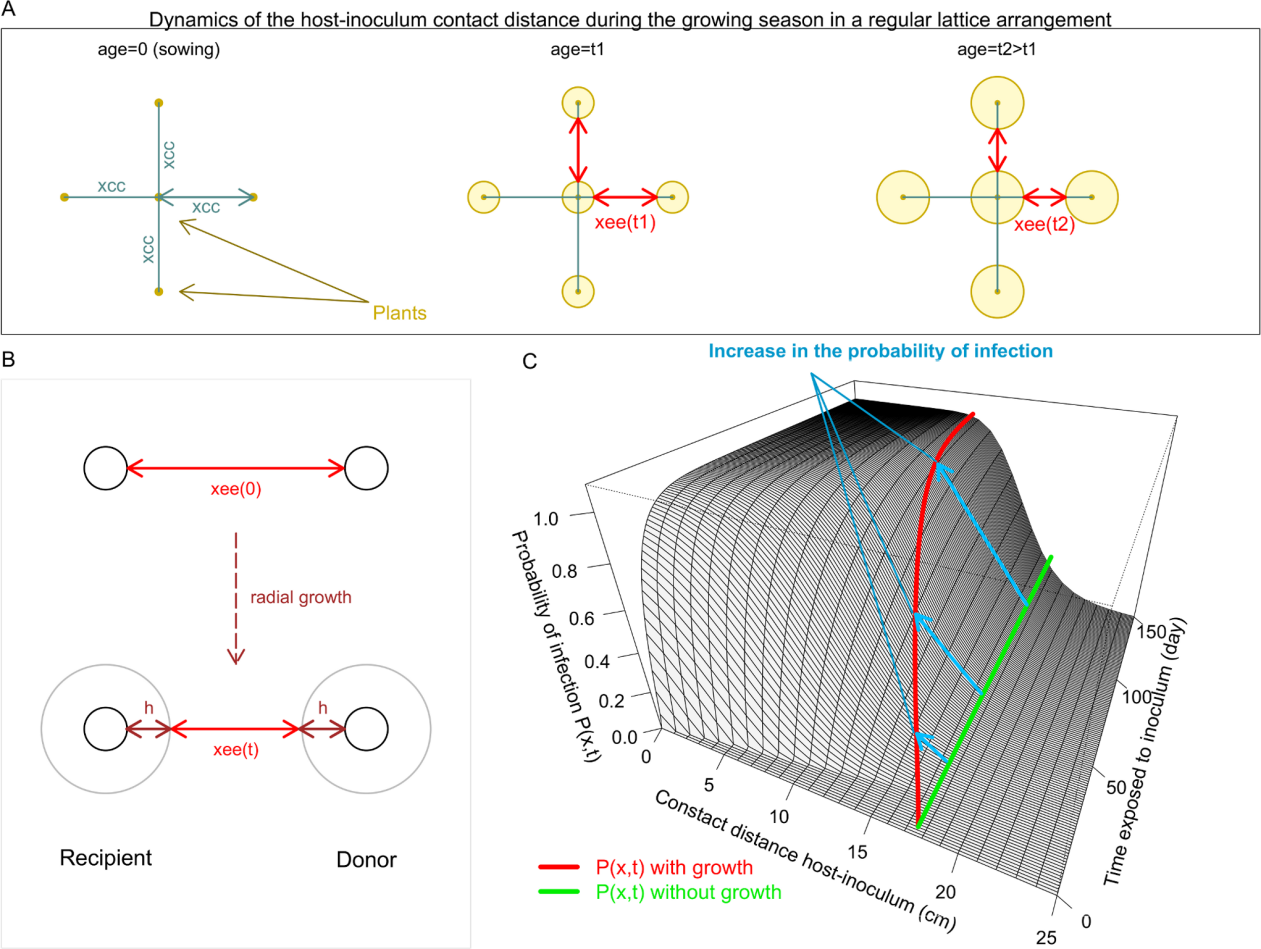
Plant growth and dynamic contact distances. A the dynamic contact distance between the tissues of neighbouring plants in a crop population: the initial distance at sowing is the centre-centre distance, x_cc_; as plants grow the edge-edge distance x_ee_ decreases; B radial growth of plants that form a pathogen donor-recipient pair; C host growth can increase the probability of infection at individual level.

**Figure 2:**
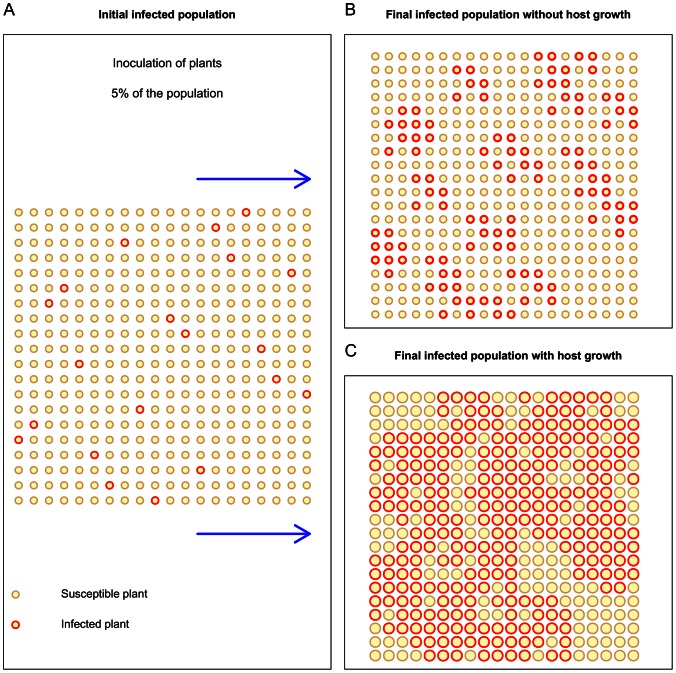
Illustration of the potential effect of individual host growth on epidemic spread. For a given initial inoculation of the population A, pathogen spread is non-invasive in the absence of host growth, i.e. infection remains localised B, whereas pathogen spread is invasive with host growth, i.e., infection spans across the population C. As plant grow, the edge-edge contact distance between hosts (larger circles) decreases, which reduces the percolation threshold distance for the system. The host population is distributed in an isotropic square lattice, with the centre of each individual located at a lattice vertice.

The spatial structure of plant populations in crop systems is usually determined by growers at the time of sowing or planting. Plant spacing, i.e. the distance (*x_cc_*) between the centres of neighbouring plants (Fig. 1A), is usually chosen in order to optimise the quality (plant shape) and yield of the cultivated crops. During the growing season, at least in non-perennial crops, the growth of individual plants reduces the *contact distance* between the tissues of neighbouring plants, which we represent via the *edge-edge* distance (*x_ee_*) (Fig. 1A). To our knowledge, this factor has not been included in previous modelling studies of soilborne epidemics, for example [22], [23], [24], where the host contact distance was represented by the static spacing *x_cc_* (Fig. 1A). Here, we test the hypothesis that the below-ground growth of host plants can, under otherwise non-invasive conditions, cause a resident soil-borne pathogen population to grow to epidemic scale and severely damage a crop during a growing season (Fig. 2); conversely, under already invasive conditions, root growth increases the disease level during the growing season. We test this hypothesis in two stages. First, we develop novel experiments on the pathozone of a plant-host soilborne-pathogen system. Second, we use these individual-level observations to parameterise a population model and simulate pathogen spread in a plant host population, contrasting models that allow for plant growth with those that do not.

Soilborne pathogens spread below-ground and, thus, it is difficult to assess the extent of their dispersal in natural and cultivated soils [25]. Generally, infection of a plant host can occur through acquisition of inoculum resident in the soil, and originating from external sources or decayed infected material, or through the spread of the fungus from living infected hosts. These two processes of infection are usually referred to primary and secondary infection. The host-centred concept of *pathozone* [26] characterises host vulnerability to infection by soilborne pathogens [22], [27] and relates to the inoculum-centred concept of *dispersal kernel* [28], [29]. The pathozone of a pathosystem is the zone around a host where an inoculation could cause infection; it is represented by a surface, *P(x,t)*, of the probability of infection of a recipient host in terms of its distance *x* to the inoculum donor and the time *t* since exposure to inoculum (Fig. 1B & 1C). In general, estimation of dispersal kernels requires epidemic data and intensive Bayesian techniques for dealing with missing data because each observation is not traceable to a specific inoculum source, e.g., [30]. The advantage of a pathozone in relation to a dispersal kernel is that it is more readily measurable, at least for soilborne plant pathogens. Measurements can be made, for example, in replicated experiments where donor (inoculum) recipient (host) pairs are placed at differing centre-centre distances (*x_cc_*) and the time of infection (if any) since host exposure is recorded. We extend the pathozone model [26] such that the donor-recipient contact distance is that between host tissue and inoculum, which we represent by the edge-edge distance *x_ee_* (Fig. 1A & 1B). As hosts grow and this distance decreases, we expect an increase in the probability of infection within given a donor-recipient pair, which associates with a change in a cross section of the pathozone surface (Fig. 1B & 1C).

Saprotrophic fungi play a central role in ecosystems function by decomposing non-living organic matter; some are also able to parasite living plants and damage crops. Experimental studies show that colony expansion of these fungi depends mainly on the endogenous supply and translocation of nutrients within a mycelial network, and on its growth strategy for given spacing among nutrient sources [17], [31], [32]. In real soils, however, this process is still poorly understood [33]. Here, we present novel results on fungal spread in cultivated soil in field conditions.

In order to investigate the influence of plant growth on the spread of a soilborne plant pathogen, we consider the parasitic fungus *Rhizoctonia solani* AG2-2IIIB on sugar beet (*Beta vulgaris L.*) as an example system. *Rhizoctonia solani* is a saprotrophic Basidiomycete that parasites a wide range hosts [34], and has been studied in controlled conditions [22], [32], [35], [36]. *Rhizoctonia solani* AG2-2IIIB, in particular, is prevalent in agricultural systems and causes brown root (crown rot disease) on sugar beet [37], [38]. We measured the pathozone of this system in field conditions and used these measurements to parameterise an individual-based population model of the development of epidemics. We show that host growth can induce a switch from non-invasive (localised) to invasive (system-wide) spread of the pathogen during a crop season (Fig. 2). We conclude the paper by discussing the importance of considering the root growth of hosts in predicting epidemic behaviour of locally-spreading pathogens, and in agronomic design for the prevention and management of plant diseases.

## Materials and Methods

### Pathosystem

In this study we considered the saprotrophic fungus *Rhizoctonia solani* anastomosis group (AG) 2-2 IIIB (isolate G6), which parasitizes a variety of crops including sugar beet, rice, maize, and ginger. As *R. solani* AG2-2 IIIB has a maximal growth rate around 30°C [39] its activity is often negligible during the early stages of a crop, when environmental conditions are cool. Although sugar beet does not exhibit a significant change in susceptibility with age, this strain of *R. solani* is known to spread late on mature plants [37], hence early infections are rare.

### Inoculum Production

Infested barley seeds were used to emulate soil residual inoculum of *R. solani*. First, barley seeds were soaked with water before autoclaving (2×1h) at 115°C, with a 24h interval between autoclaving. Then the autoclaved barley was inoculated with mycelial plugs removed from the margins of 7 days old colonies grown on malt agar at 20°C. Finally, the inoculated seeds were incubated for 3 weeks at 20°C.

### Pathozone: Placement Experiment

The probabilities of infection within pairs of inoculum-donor and host-recipient were measured in field experiments, each with one of two types of pathogen inoculum, primary inoculum or secondary inoculum, which provided low (limited in time) and high (unlimited in time) nutrient levels for mycelial expansion of the fungus, respectively. In each experiment, primary inoculum consisted of five infested barley seeds, while secondary inoculum was sugar beet inoculated four weeks in advance and exhibiting at least 50% of diseased root surface.

Field experiments were carried out at Le Rheu, France (UE787 Unité Expérimentale de la Motte, coordinates 48°06′ N, 1°48′ W) in 2011 with the permission of the Inra Experimental unit UE787. At this site the soil is silty, with mean pH value of 6.69, total nitrogen content of 1.30 g/kg, organic matter content of 19.14%, and cation exchange capacity of 71.68 cmol(+)/kg. Soil analyses at five points in the area of the experiments revealed low global variability in physicochemical parameters. The sugar beet crop (cv Skipper) was sown on April 5^th^ 2011 using a pneumatic drill. The crop was irrigated to prevent soil dehydration, and was managed according to the common farming practice except that no fungicide was applied. As *R. solani* had not been introduced and sugar beet had not been grown in this field previously, we assumed that the soil was free of inoculum before the experiment. In order to prevent undesirable infections between inoculum-donors and host-recipients belonging to distinct pairs, we kept plant spacing above 80 cm by thinning out the crop manually.

The probability of infection *P(x,t)* for each given distance x and time t since exposure (Fig. 1C), was obtained by assessing, via destructive sampling, how many inoculations had caused infection. The experiment was repeated for a range of x and t values, and for each value of x and t comprised *n_tot_* = 25 replicate pairs of inoculum-donor and host-recipient. Primary-inoculum donors were placed at six ‘contact’ distances *x_ee_ = *0, 2, 4, 6, 8 and 12 cm from the edge of 93-day-old plant-recipients, while secondary-inoculum donors were placed at six contact distances *x_ee_* = 0, 5, 10, 15, 20 and 50 cm from the edge of 99-day-old plant-recipients. The use of larger contact distances for pairs involving secondary rather than primary inoculum is supported by laboratory experiments with *R. solani* that suggested secondary inoculum had further range than primary inoculum [23]. For primary inoculum, and for each of the six ‘contact’ distances *x_ee_*, root infection status was assessed (destructively) in distinct replicate pairs after four different periods of exposure to inoculum: 14, 20, 29 and 40 days. For secondary inoculum, destructive assessment of infection status was carried out gradually across the ‘contact’ distances, starting with the replicates with *x_ee_* = 0 through to those with *x_ee_* = 50 cm, at the above periods of exposure to inoculum, until finding the first distance with a zero count of infection; we assumed a zero probability of infection for the remaining larger distances. We obtained measures of the probability of infection for each distance *x_ee_* after 8, 14, 23 and 42 days of inoculum exposure, and additional points for *x_ee_* = 15, 20 and 50 cm and *x_ee_* = 20 and 50 cm after 55 and 62 days of exposure, respectively.

### Parameters for the Population Model

Following Kleczkowski et al. (1997) [23], we built a model for the rates of primary (*β_p_*) and secondary (*β_s_*) infection of a recipient host in a donor-recipient pair, by compounding the diminishing effects of several biological processes on a basic (maximum) rate:
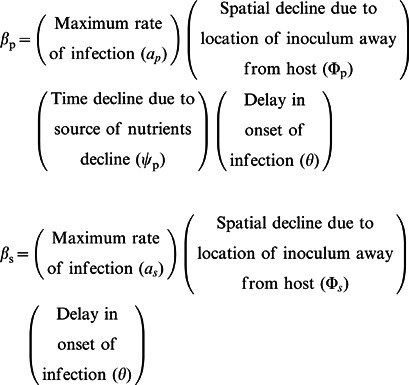
(1)


Assuming that these processes operate independently (at least within a single season, as in this study), then the rates at given distance *x* between the inoculum donor and host recipient, and at time *t* of exposure of the recipient, are given by a multiplication of functions of either distance or time:
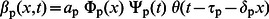
(2)

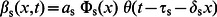
(3)where *a_p_* and *a_s_* are the maximum rates of infection, and the delay in onset of infection is described by a step function, *θ(t) = 0* if *t<0* and *θ(t) = 1* if *t>0*. The parameters *τ_p_* and *τ_s_* represent initial delays in fungal infection, and *δ_p_* and *δ_s_* allow for increases in the delays with distance *x* (see Table 1). For the spatial components of these rates we use Gaussian functions [40]:

**Table 1:** Parameters of the model, interpretation, estimated distributions.

Parameter	Interpretation	Units	Mean	SD	q-2.5%	Median	q-97.5%
secondary inoculum							
a_s_	maximum rate of infection	d^−1^	0.134	0.015	0.106	0.134	0.165
σ_s_	rate of spatial decline	cm^−2^	0.01391 (1/72)	0.00117	0.01181	0.01384	0.01633
δ_s_	rate of delay	d cm^−1^	0.90	0.09	0.68	0.92	1.00
τ_s_	minimum delay	d	0.47	0.41	0.001	0.36	1.55
primary inoculum							
a_p_	maximum rate of infection	d^−1^	1.79	0.84	0.75	1.6	4.4
σ_p_	rate of spatial decline	cm^−2^	0.00624 (1/160)	0.00488	0.00022	0.00507	0.01849
d_p_	rate of temporal decline	d^−1^	0.371	0.144	0.202	0.338	0.859
δ_p_	rate of delay	d cm^−1^	0.85	0.16	0.40	0.90	0.99
τ_p_	minimum delay	d	5.70	2.37	0.20	6.32	9.33



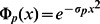
(4)

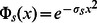
(5)where *σ_p_* and *σ_s_* are rates of spatial decline of the probabilities of primary and secondary infection, respectively. For primary infections, we assumed there is a limited initial source of nutrient that decline exponentially with time as the fungus uses nutrient for growing and exploring the soil (nutrient exhaustion):
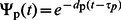
(6)where dp is the rate of temporal decline of probability of primary infection, and τp is defined above (see Table 1).

Contrary to other anastomosis groups of *R. solani* (AG4, for example), AG2-2 IIIB is known to parasitize mature sugar beets [34], which do not exhibit a significant change in susceptibility with age. As a mature sugar beet plant contains a large amount of nutrient, we considered that an infected plant provides an unlimited source of nutrient to mycelium, and thus that fungal growth is not limited in time by nutrient collapse. In other words, we assumed that in the experiment there was no temporal decline neither in the infectiousness of donor hosts (equation (3)) nor in the susceptibility of recipient hosts.

The pathozones for primary and secondary infection of a recipient host are represented by probability distributions (*P_p_* and *P_s_*) over distance *x* (from the location of the host) and time *t* (after exposure of the host), that obey the equations:
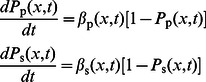
(7)


Solving equations (7) together with the assumptions (2)-(6), gives the explicit model for the dynamics of the pathozones:



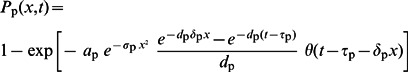
(8)




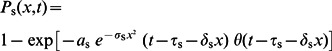
(9)where, *t* is the time since exposure of the host to the inoculum, and, for a pathozone that does not incorporate host growth [26], *x* is the static contact distance between the host and the inoculum.

We assessed and parameterised the pathozone models (8)–(9) by fitting the experimental pathozone data with the following random process, describing the number of replicated donor-recipient experiments in which there was an infection:
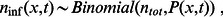
(10)where *P = P*_p_ or *P*_s_ is the probability of a single infection given by (8) or (9). Specifically, we fitted *n_inf_* to the number of infections, among *n_tot_* = 25 replicates, for each donor-recipient distance *x* and period of exposure *t.* We implemented this estimation of the pathozone parameters in (8)–(9) via Bayesian Markov Chain Monte Carlo sampling with likelihood function based on (10) and non-informative prior distributions, run in OpenBugs [41] and with posterior densities analysed in R [42]. We verified the consistency of the models with the data observations by checking that the observations of the probability of infection P(x,t) are contained within the posterior predictive distribution of the fitted pathozone models (8)–(9) (Appendix S1).

We used the estimated parameters of the pathozone models (8)–(9) to parameterise the rates of infection (2)–(3) in population models of pathogen spread. In these population models, we incorporated host growth by using a dynamic model for the contact distance between a given host and inoculum inferred from data as described below.

### Plant Growth and Dynamic Distances between Hosts

The radial growth of belowground parts of sugar beet was assumed to be spatially isotropic and homogeneous in the soil across the field. We considered the radius of the tuberous root at the neck of plants to be a measure of their radial size. We described the increment in the radius of the host root system, *h(t)* (Fig. 1B), by a logistic equation with asymptote 5 cm (see Appendix S2 for supporting data and parameterisation).
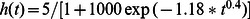
(11)


The growth of inoculum-donor sugar beet can be affected by *R. solani* infections in different ways. If an infection occurs when the host is still small, the fungus colonizes the root system rapidly and kills the host. In a mature plant, the root system is more developed and the host can survive pathogen colonization longer by producing new roots [43]. We use a simple model, inspired by our qualitative observations, for the dynamics of the edge-edge spacing *x_ee_* in a donor-recipient pair that incorporates these differences in growth of infected sugar beet, by allowing for a variable contribution from the growth of the inoculum-donor host according to its age at the time of infection of the recipient host, *t_inf_*:
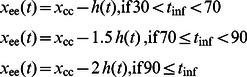
(12)where *x_cc_* is the (static) distance between host centres (Fig. 2C). Changes in the probability of infection induced by host growth, which we describe using the model (11)–(12) for the contact distance between host and inoculum (Fig. 1B), are obtained by replacing *x* in (8)–(9) with *x_ee_(t)* in (12).

### Spatial Population Model and its Simulation

For many soilborne diseases, pathogen spread is localised and, for a population distributed on a lattice, occurs predominantly between nearest neighbour plants, e.g., within a von Neumann neighbourhood of the inoculum sources [13], [44]. In order to investigate the effect that host growth, which reduces the contact distance between nearest neighbours, has on epidemic behaviour, we implemented a spatially-explicit host population model. Specifically, we used an individual-based stochastic model of the spread of *R. solani* between nearest-neighbour plants, where individuals are arranged in a square lattice, and each individual can be either Susceptible (*S*) or Infected (*I*). We compared the spatial spread of *R. solani* between two versions of this model that differ in the specification of the contact distance between neighbouring hosts. In one version of the model, this distance is static and equal to the centre-centre spacing of hosts *x_cc_* (the edge-edge distance *x_ee_* at the time of crop sowing); this is the commonly used approach for soilborne plant diseases. In another version of the model, the neighbour contact distance is dynamic and given by the edge-edge distance *x_ee_(t)* (Fig. 1A and equation (12)). We simulated 1000 continuous-time Markov chains on a 30 by 30 square lattice (i.e., a population with 900 hosts) using a *first event* algorithm (Fig. 2) [45]. As *R. solani* AG2-2 IIIB is known to initiate epidemics late, we assumed that the pathogen started spreading 30 days after sowing (*t_inf_* >30 days). Simulations were stopped 250 days after sowing, which corresponds to a typical sugar beet growing season.

For our purpose of demonstrating the impact of host growth on epidemic development, we focused on the effect of host growth on secondary infection and ignored its effect on primary infection. This choice is justified because the spatial distribution of resident primary inoculum is usually unknown or not manageable, while the spacing of hosts (*x_cc_*) can be designed by farmers in order to account for plant growth. We considered an initial random distribution of primary infection in 5% of the host population (Fig. 2), representing, for example, infected imported seed. In our model, the probability that a Susceptible plant becomes Infected during a time interval *[t,t+dt]* is given by:
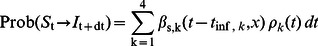
(13)where *ρ_k_(t) = 1* if neighbour *k* is infected at time *t* and *ρ_k_(t) = 0* otherwise, *β_s_* is the rate of secondary infection (equations (3)–(6)), t*_inf,k_* is the time of infection of nearest-neighbour *k*, and distance *x* is the same for every neighbour (x* = x_cc_* or *x_ee_(t)*, equation (12)). The parameters of the function *β_s,k_* are those estimated for the pathozone *P(x,t)* in equation (9); specifically, we used the means of the corresponding Bayesian posterior distributions.

According to the asymptotic radius of sugar beet (5 cm, Appendix S2) the spacing of plant centres *x_cc_* has to be greater than 10 cm. In order to assess the effects of host growth on pathogen invasion for differing initial plant spacing x_cc_, we simulated epidemics for *x_cc_* = 11, 14 and 17 cm. Then we compared the spread of *R. solani* among population models where the contact distance between neighbour hosts is static and where it is dynamic.

## Results

### Pathozones: Infection at Individual Level

**Figure 3:**
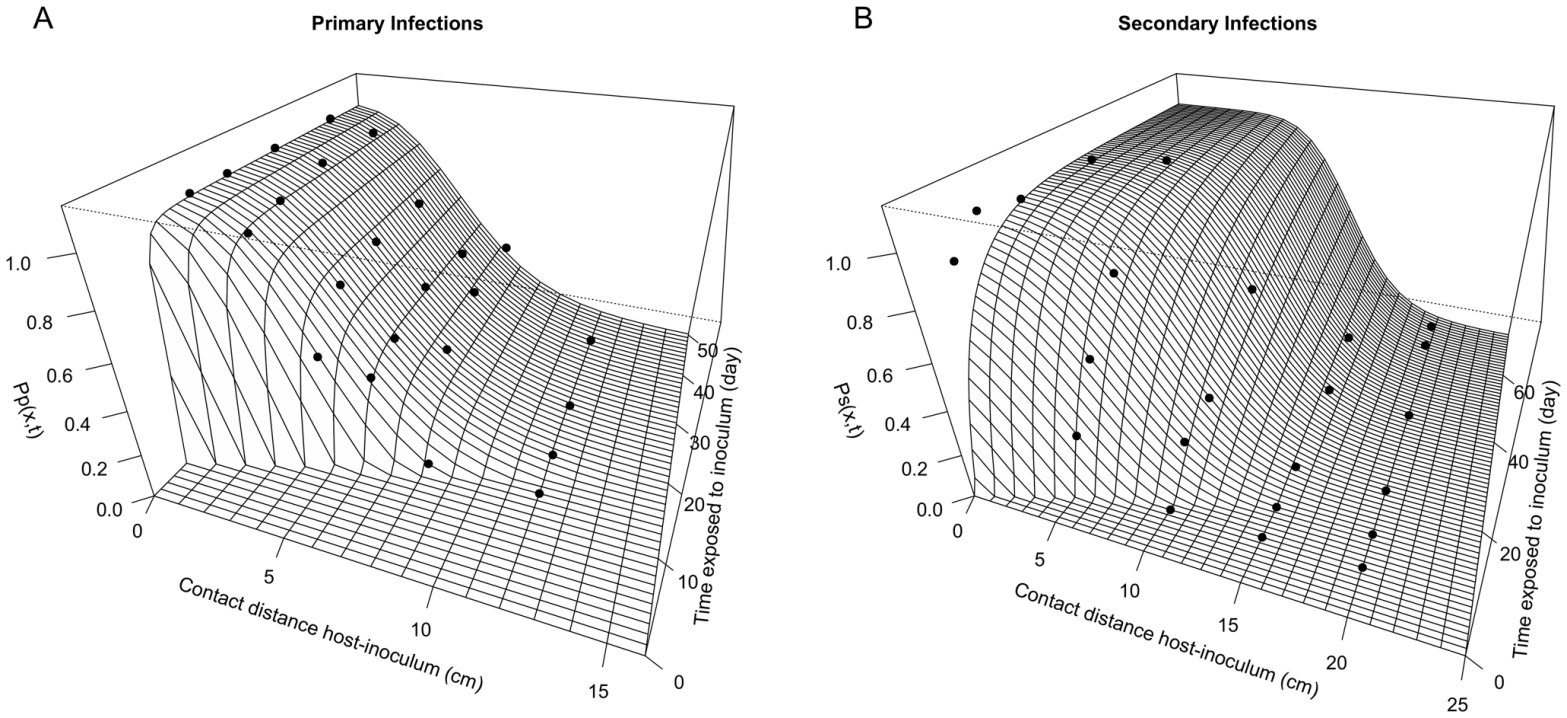
Pathozone measurements. Probabilities that an inoculum-donor placed at a certain distance from a host-recipient infects the recipient susceptible plant after a given time of exposure (see Fig 2). A Primary infection (caused by infected seeds) and B Secondary infection (caused by infected hosts). Points correspond to data obtained in placement experiments. In B, points at 50 cm are not shown but the corresponding counts are zero and fit the function well.

The pathozone models (8) and (9) captured the essential pattern of the pathozone data (Fig. 3 & Appendix S1). The estimated time delays in infections, parameters *τ_p_* and *τ_s_*_,_ reveal differences between the primary and secondary infection profiles despite having considerable uncertainty (Table 1). The estimated median delay is higher for primary infection than for secondary infection (5.7 and 0.5 day, respectively) and the corresponding confidence ranges do not overlap (Table 1). For secondary infections, the estimates of most parameters (*a_s_, σ_s_, δ_s_*) have low uncertainty. For primary infection, the estimates of the spatial (*σ_p_*) and temporal (*d_p_*) decline rates show significant uncertainty, whereas the estimates of the delay (*δ_p_*) and maximum rate (*a_p_*) have low uncertainty. The local discrepancy (Fig. 3B) between the data and the pathozone model in the confined region of small host-inoculum contact distance and small time of exposure may suggest there was particularly large variability in the infection process in this region (see Appendix S1). These observations were, nonetheless, contained within the uncertainty of the fitted model (Appendix S1). However, this localised discrepancy may result from experimental error (e.g., difficulty in assessing infection at early plant necrosis), or a need to relax some of the model assumptions, e.g., a non-Gaussian spatial decline or a delay parameter in (6) independent from that in (2).

The distance between inoculum donor and host recipient strongly influenced the probability of infection (Fig. 3). In fact, the transmission of infection was limited to a contact distance of 12 cm for primary inoculum and 20 cm for secondary inoculum. Contrary to primary infection, the probability of secondary infection displayed a long-term plateau close to 1 for short distances, up to 5 cm, and increased slowly with time for distances up to 15 cm. The ‘scale’ of spatial decline was smaller for secondary infection than for primary infection (1/√σ_s_ = 1/√72 and 1/√σ_p_ = 1/√160, respectively), which is reflected in a sharper decline with distance *x* (after the plateau) in *P_s_* than in *P_p_*.

### Epidemics: Plant Growth and Pathogen Invasion

**Figure 4:**
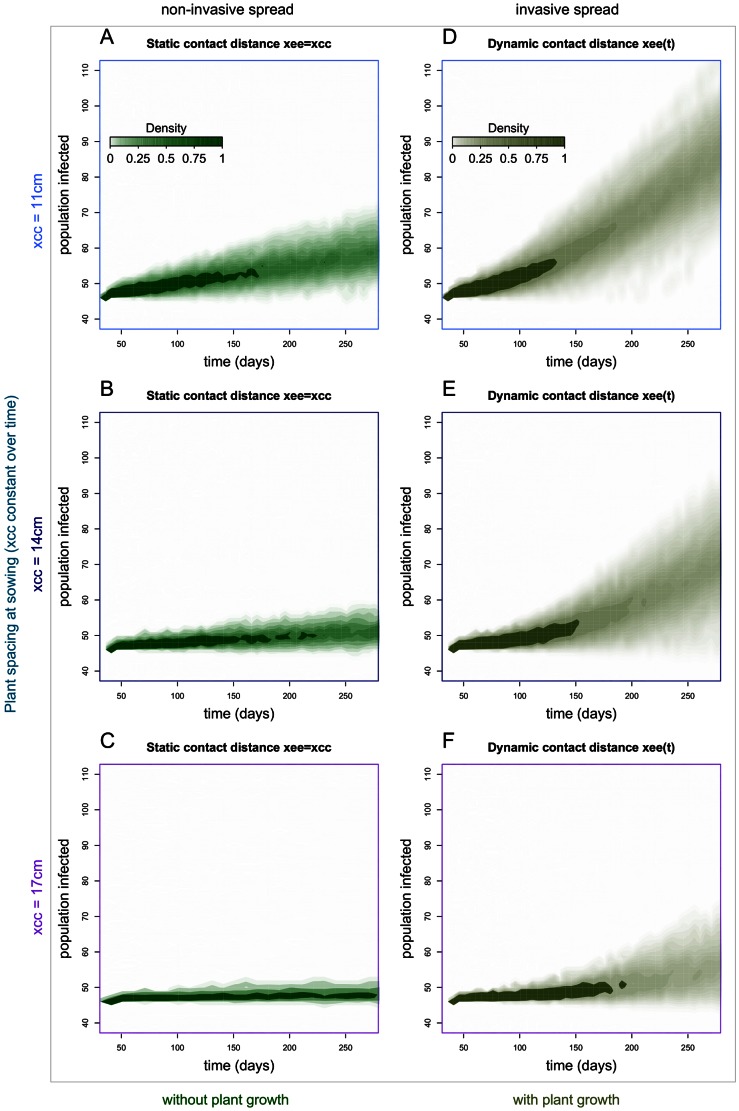
Pathogen invasion of a host population in spatially-explicit models with and without host growth. Predicted distribution of epidemic outcomes, (A–C) with a static host contact (edge-edge) distance equal to plant spacing at sowing, and (D–F) with dynamic host contact (edge-edge) distance. Upper, middle and lower graphs are simulation outcomes for distance x_cc_ = 11, 14 and 17 cm, respectively. Each graph shows the density of 1000 simulations ran on a 30*30 square lattice (900 individuals) with 5% of individuals inoculated 30 days after sowing (age = 0). The shading density represents the proportion of simulations that associate with each point on the graphs.

Changing host density by changing the initial plant spacing *x_cc_* affected the spread of *R. solani* within the regularly-spaced host population: an increase in x_cc_ led to a decrease in the final size of the simulated epidemics (Fig. 4). In the model with static contact distance between host tissues there was no epidemic take-off, as shown by the lack of a trend that rises non-linearly because of secondary infection (Fig. 4A–C & Appendix S3). With the smaller x_cc_, the part of the population infected raised very slowly but linearly (see Appendix S3) until the end of the crop season (Fig. 4A). With the larger x_cc_ the part of the population infected reached an early asymptote, but one that is well below the host population capacity, which is typical of non-percolating or non-invasive spread.

In the model with host growth, and thus with decreasing contact distance, the systems with *x_cc_* = 11, 14, and 17 cm switched to invasive spread towards the population capacity of 900 hosts (Fig. 4D–4F), as shown by the wider distributions of epidemic size and their non-linear trend (see Appendix S3). Prior to take-off, the trajectories of simulated replicate epidemics were narrowly spread (darker shades); after take-off, the trajectories spread widely away from each other and the range of their distribution increased with time in an accelerated way. For *x_cc_* = 14 and 17 cm, the distribution of the number of infected hosts is bimodal, with a narrow branch of realisations in which the pathogen is not non-invasive (lower part of figures 4E–4F).

## Discussion

Considering *R. solani*-sugar beet as an example pathosystem, we have shown, using experimental and modelling approaches, that the expansion of plant roots, which reduces the spacing between neighbouring plant tissues, can trigger the development of soilborne pathogen epidemics. For systems in conditions short of their epidemic threshold, host growth can cause a transition from non-invasive (patchy) to invasive (system-wide); conversely, for systems in epidemic conditions, host growth can enhance epidemic development (Fig. 2 & 4). Albeit the effect of host density and spatial distribution on plant pathogen invasions has been studied [12], [14], [19], to our knowledge the effect of radial host growth on epidemic behaviour has not been addressed in modelling studies. In this paper we have used models that scaled-up behaviour from individual level (e.g. pathozone) to population level (epidemic). At individual level, the cryptic decrease in the contact distance between host and inoculum, caused by the expansion of belowground parts of plants such as roots, leads to an increase in the probability of pathogen transmission (Fig. 1). At population level, our results exemplify the occurrence of a percolation transition [16], [17] due to temporal change in local pathogen transmission.

As parasites impose an energy cost upon their hosts, they generally induce a decrease in host growth. However, there are instances where infection by a plant [46] or animal [47], [48] pathogen is associated with an enhancement in host growth. For plant-parasite systems, it has been shown that infection by some fungal soilborne pathogens can enhance belowground host growth [43] through the production of new roots on healthy parts of the root system, which allows plants to counterbalance root surface loss due to necrosis. Our results suggest that, where diseases-induced root growth does occur, it has the potential to increase pathogen transmission and trigger invasions of locally-dispersing plant pathogens. Hence, a root-growth physiological response of plants to microbial parasitism would benefit the pathogen population; therefore, from co-evolutionary perspective it can be viewed as a manipulation of the host by a parasitic pathogen. This hypothesis regarding soilborne plant pathogens, relates to the findings of a previous modelling study, which demonstrated that when infection occurs at short distance, between nearest-neighbour but mobile hosts, parasites always gain from an increase in their host’s rate of movement [49].

Host density is an important factor in the epidemiology of plant diseases [12], [14], [18], [19]. In this work we have shown that, in addition to the number of hosts present in a given area, changes in the density of susceptible tissue are also important and can cause, for example, a decrease in the contact distance between contiguous plants (Fig. 2 & 4). Our findings suggest that practitioners of agriculture and arboriculture should account for the growth of host roots in the design and management of crops in order to reduce the risk of soilborne disease epidemics in healthy crops, and losses in yield and quality in crops where epidemics occur. In theory, one would choose crop geometries (plant spacing and arrangement) at sowing or planting that ensure the spacing between neighbouring plants roots remains above a critical threshold for epidemic occurrence before harvest. Estimating such a threshold distance for a given pathosystem may be difficult, e.g., it would depend on how far fungal hyphae can bridge the space between plant roots. Hence, it may be desirable to set plant spacing at least twice as large as the perceived range of root growth. However, it is still challenging to predict root system expansion in soils [50] because of the number of factors that affect the plastic growth of roots [51]. For plants exhibiting taproot systems (e.g. sugar beet, carrot, radish) it may be doable to manage plant spacing. However, a decrease in host density could cause economic loss through a reduction in crop yield and quality (e.g. changes in plant shape), and an increase in the effort to manage weeds that develop in empty spaces. It would be a useful goal to develop models for the design of optimal plant-spacing that integrate disease prevention via reduced plant density, profit from yield, management costs, and environmental impact from intensive farming, for a range of agricultural crops. Most crop plants with an adventitious root system (e.g. wheat, barley, maize) will tend to fill in gaps, so modest reductions in density may not be efficacious in reducing disease risk. One solution that may apply to several systems would be to intertwine host and non-host plants and benefit from inter-specific plant competition, which would reduce expansion of the host plant root system expansion. Crop variety mixtures are a cultural practice successfully tested for increased resistance to diseases [52], [53]. For example, it has been shown in highly controlled environments, that soilborne pathogen transmission is reduced in mixed populations of young hosts and resistant plants [54]. Growers design crop systems according to agronomic criteria which involve crop physiological traits and practical issues; but it is unusual to account for epidemiological parameters such as threshold distances. Crop mixing is recognized as a useful practice for disease management [52], [53], and for soilborne disease without waterborne transmission, they may allow practitioners to keep host plants above their threshold contact distance while adding value to the extra free space by simultaneously growing non-susceptible plants. The design of crop system is complicated process where growers and agronomists have to make important choices on crop species and technical practices according to their knowledge in order to ensure a reasonable income in an uncertain future (bad weather, market prices, disease). Albeit, the use of crop mixing could involve some practical difficulties and new ecological knowledge to optimise their production, this practice would permit to create more resilient exploited plant systems towards soilborne pathogen invasions [52], [55]. However, following results of Otten et al. (2005) it may be important to assess the long term effects of mixed-population on the selection of quantitative traits of soilborne pathogens [56] to adapt mixtures to pathogen evolution.

In this study we considered a strain of the saprotrophic fungal pathogen *R. solani* that causes substantial damage in agriculture [5], [37]. Our measurements of the pathozone of *R. solani* in fields conditions are novel, as previous studies were made in controlled and non-soil conditions [22], [23], [35]. The mycelial spread of saprotrophic fungi in real soils is still poorly understood [33], in particular because the spatial and temporal heterogeneity of soils makes them complex environments. Albeit, despite the specificity of our system, it is likely that our results are transferable to other soils; similar experimentation in differing soils would enable testing the generality of these results.

Our experiments contrasted pathozone behaviour when differing levels of nutrient are available for saprotrophic mycelial growth. Indeed, while secondary inoculum (from an infected plant) supplied a large amount of nutrient, primary inoculum (from infested barley seeds) provided a relatively small level of nutrient. As previously demonstrated in microcosm experiments [23], the probability of infection of a plant was greater when the mycelium introduced about the host had access to a larger source of nutrients (Fig.3). This trend can be explained by the ability of the fungus to translocate nutrients from one area of the mycelium to another, a well known process in Basidiomycetes fungi [31], [33], [36]. In the case of primary inoculum, the absence of a plateau and the small spatial extent of the pathozone (Fig. 3A) suggest there was a low hyphal density or a low infectivity, and a poor capacity of *R. solani* to uptake nutrients directly from the soil matrix. In the case of the pathozone of secondary inoculum (Fig. 3B), its spatial extent suggests the fungus has the ability to translocate nutrients up to 20 cm, while its plateaux at shorter distances suggests there was strong infectivity and a high hyphal density about the inoculum. From an epidemiological point of view, these observations are particularly important for understanding pathogen spread through secondary infection aided by the level of nutrient available in the host.

In demonstrating the effect of radial root expansion on the development of epidemics using a population model we made important assumptions that we now discuss. First we did not consider primary infections from inoculum resident in the soil because the distribution of residual inocula is usually heterogeneous and cryptic, and, therefore, difficult to manage. As differences in geometrical arrangement and germinability of inocula particulate can induce small differences in initial infections, taking into account primary infections might have led to an increase in the variability among replicates epidemic trajectories [23]. Second, as the assessment of plastic root expansion is technically challenging, we adopted the above-ground radius at the neck of the tuberous root of sugar beet as a measure of the belowground parts of the plants, and thus we underestimated the extent of the root system (Appendix S2). Considering a more realistic measure of the root system could have further amplified the effect of host growth on pathogen invasion. Third, during field experimentation we have assumed that the contact distance between inoculum-donor and host recipient was static and thus we have neglected the growth of healthy (recipient) and infected (donor) plants. In experiments involving secondary inoculum, the transplantation of the infected donor plants, which were already weakened by the disease, destroyed their secondary root system. Hence, although these plants survived transplantation their subsequent radial growth (h, for ‘donor’ in Fig. 1B) was negligible. As the experiments were run on mature plants (>90 days) the radial growth of host-recipients (h, for ‘recipient’ in Fig. 1B) was also small and close to the measurement error, estimated at 0.5 cm and 1.5 cm for pair experiments involving primary and secondary inoculum, respectively. Tackling plant growth in placement experiments is a recurrent difficulty; however, this was not a major issue in our experiments.

## Supporting Information

Appendix S1 Pathozones’ uncertainty and observations.
(DOC)

Appendix S2 Sugar beet growth.
(DOC)

Appendix S3 Trends in model outcomes.
(DOC)
