# Supplementary material for: Host Growth Can Cause Invasive Spread of Crops by Soilborne Pathogens
Source: PLoS One. 2013 May 8;8(5):e63003. doi: 10.1371/journal.pone.0063003 (PMC3648505; doi:10.1371/journal.pone.0063003)
Supplement: Appendix S1 — Pathozones’ uncertainty and observations. (DOC) [file pone.0063003.s001.doc]

Appendix S1: Pathozones’ uncertainty and observations

**Methodology**

The consistency of mechanistic models describing the spatio-temporal process of infection at individual level (i.e. pathozone behaviour) in the cases of primary (s1.1) and secondary (s1.2) infection was assessed by checking that the experimental observations were contained within the posterior predictive distributions of the fitted pathozone models.

$P_{p}(x,t)=1-\exp\left[- a_{p} e^{-\sigma_{p}x^{2}} \frac{e^{-d_{p}\delta_{p}x}-e^{-d_{p}(t-\tau_{p})}}{d_{p}} \theta(t-\tau_{p}-\delta_{p}x)\right]$ (s1.1)

$P_{s}(x,t)=1-\exp\left[-a_{s} e^{-\sigma_{s}x^{2}} (t-\tau_{s}-\delta_{s}x) \theta(t-\tau_{s}-\delta_{s}x)\right]$ (s1.2)

*A-posteriori* distributions of the probability of infection were obtained by simulating the model with parameters sampled from the posterior distributions of the models parameters. These parameter posterior distributions were previously obtained via Bayesian Markov Chain Monte Carlo sampling (Gibbs sampling) using a likelihood function based on (s1.3) and the experimental data, and non-informative prior distributions of the models parameters. Posterior distributions of the probability of infection were represented with Box-and-Whisker Plots (Boxplot) which gave a good overview of the simulated data distributions and allowed a visual check against the experimental data and its potential outliers.

(s1.3)

**Results**

***Primary infection***

Figure S1.1 Posterior pathozone profile for primary infection. *A posteriori* distributions of probabilities P_p_(x,t) that an inoculum-donor (i.e. 5 infested barley seeds) placed at a certain contact distance (x=0, 1, 2, 4, 6, 8, 10, 12 and 14 cm) from a host-recipient infects the recipient susceptible plant after a given time of exposure (t = 14 (A), 20 (B), 29 (C) and 42 (D) days). Posterior distributions, obtained with Markov Chain Monte Carlo sampling (1000 iterations) and posterior distributions of model (s1.1) parameters, are shown with boxplots (blue) where the end of dashed lines represents minimum (bottom) and maximum (top) quartiles that exclude outliers (blue empty circles). Observed probabilities of infection (x=0, 2, 4, 6, 8 and 12 cm) are represented by red full points.

***Secondary infection***

Figure S1.2 Posterior pathozone profile for secondary infection. *A posteriori* distributions of probabilities P_s_(x,t) that an inoculum-donor (i.e. an infectious plant) placed at a certain contact distance (x=0, 2, 5, 7, 10, 12, 15, 17, 20 and 25 cm) from a host-recipient infects the recipient susceptible plant after a given time of exposure (t = 8 (A), 14 (B), 23 (C) and 42 (D) days). Posterior distributions, obtained with Markov Chain Monte Carlo sampling (1000 iterations) and posterior distributions of model (s1.2) parameters, are shown with boxplots (blue) where the end of dashed lines represents minimum (bottom) and maximum (top) quartiles that exclude outliers (blue empty circles). Observed probabilities of infection (x=0, 5, 10, 15 and 20 cm) are represented by red full points.
