# Supplementary material for: Host Growth Can Cause Invasive Spread of Crops by Soilborne Pathogens
Source: PLoS One. 2013 May 8;8(5):e63003. doi: 10.1371/journal.pone.0063003 (PMC3648505; doi:10.1371/journal.pone.0063003)
Supplement: Appendix S2 — Sugar beet growth. (DOC) [file pone.0063003.s002.doc]

Appendix S2: Sugar beet growth

*Data*

To describe the radial growth of sugar beet we use data provided by the *Institut Technique français de la Betterave industrielle* (ITB). The dataset consisted of daily measures of the radius of 63 plants grown in field conditions. Measures were obtained using a Pepista tool (AgroRessources, France) and data were collected between the ages of 79 days and 197 days after sowing.

*Model parameterisation*

First a simple logistic equation (s2.1) was fitted to data using least-squares. This model captured the main behaviour of the increment in the radius h(t) after 79 days, for which data were available. Nevertheless, the model did not seem realistic enough for early ages. In order to introduce more realism in this model, we refitted a more general model in which we fixed two parameters to impose the same asymptote obtained previously (5 cm) and an initial value close to zero.

$h(t)=a/(1+b.e^{-c.t})$ (s2.1)

$h(t)=50/\{1+1000.\exp(-a.t^{b})\}$ (s2.2)


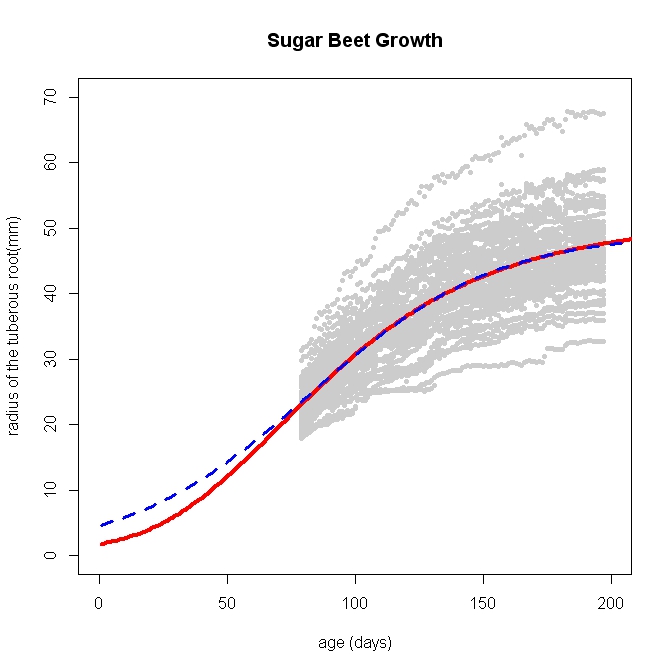


Fig. S2.1 Model for growth of the tuberous root of sugar beet at the neck. Data used for parameters estimation are represented by grey points. The blue dashed line corresponds to the first model without constraints and the red solid line is the second model used for individual-based population simulations.
