# Supplementary material for: Host Growth Can Cause Invasive Spread of Crops by Soilborne Pathogens
Source: PLoS One. 2013 May 8;8(5):e63003. doi: 10.1371/journal.pone.0063003 (PMC3648505; doi:10.1371/journal.pone.0063003)
Supplement: Appendix S3 — Trends in model outcomes. (DOC) [file pone.0063003.s003.doc]

Appendix S3: Trends in model outcomes

***Methodology***

In order to distinguish linear from non-linear trends in model outcomes, which are typical of non-invasive and invasive pathogen spread, respectively, we regressed a second order polynomial and a linear model on the number of infected hosts among replicate stochastic realisations of the population model (*Y_i_*) at a given sequence of times *t*:

$Y_{i}=\alpha_{1}+\alpha_{2}t_{i}+\alpha_{3}t_{i}^{2}+\varepsilon_{i}$ (s3.1)

$Y_{i}=\gamma_{1}+\gamma_{2}t_{i}+\varepsilon_{i}$ (s3.2)

Here ε_i_ is a standardised normal error, and the coefficients α_1_, α_2_, α_3_ and γ_1_, γ_2_ were unconstrained.

In order to focus on trends after an apparent change in epidemic behaviour due to host growth, we cut-off the data and only kept the number of infected hosts after *t>80 days, t>110 days* and *t>140 days* for *x_cc_=11, 14* and *17cm* respectively.

A non-linear trend is established when α_3_ is significantly different from zero (p-value<0.05) whereas a linear trend is recognised when α_3_ is not significant and when the trend is better described by model (s3.2) (i.e. smaller AIC and γ_1_, γ_2_ significantly different from zero).

***Results***

Table S3.1 Estimated parameters, p-value and Akaike information criterion (AIC) of the polynomial regression (equation (s3.1)) to the trend in the number of infections in the simulated stochastic population models for initial spacing *x_cc_=11, 14 and 17cm*.

|  | *α_1_* | *α_2_* | *α_3_* | *AIC* |
| --- | --- | --- | --- | --- |
|  |  |  |  |  |
| *Initial spacing = 11cm* |  |  |  |  |
|  |  |  |  |  |
| Static contact distance | 4.52 x 10^1^ (p<10^-15^) | 4.97 x 10^-2^ (p<10^-15^) | -4.81 x 10^-6^ **(p=0.67)** | 40567 |
| Dynamic contact distance | 4.16 x 10^1^ (p<10^-15^) | 7.20 x 10^-2^ (p<10^-15^) | 3.15 x 10^-4^ **(p<10^-15^)** | 191861 |
|  |  |  |  |  |
| *Initial spacing = 14cm* |  |  |  |  |
|  |  |  |  |  |
| Static contact distance | 4.73 x 10^1^ (p<10^-15^) | 6.60 x 10^-3^ (p=0.33) | 3.08 x 10^-5^ **(p=0.09)** | 9525 |
| Dynamic contact distance | 4.48 x 10^1^ (p<10^-15^) | 9.92 x 10^-3^ (p=0.27) | 2.92 x 10^-4^ **(p<10^-15^)** | 102798 |
|  |  |  |  |  |
| *Initial spacing = 17cm* |  |  |  |  |
|  |  |  |  |  |
| Static contact distance | 4.69 x 10^1^ (p<10^-15^) | 4.53 x 10^-3^ (p=0.79) | 1.75 x 10^-6^ **(p=0.97)** | 1423. |
| Dynamic contact distance | 4.58 x 10^1^ (p<10^-15^) | 2.70 x 10^-3^ (p=0.9) | 1.29 x 10^-4^ **(p<0.02)** | 21236 |
|  |  |  |  |  |

Table S3.2 Estimated parameters, p-value and Akaike information criterion (AIC) of the linear regression (equation (s3.2)) to the trend in the number of infections in the simulated stochastic population models for initial spacing *x_cc_=11, 14 and 17cm*.

|  | *γ_1_* | *γ_2_* | *AIC* |
| --- | --- | --- | --- |
|  |  |  |  |
| *Initial spacing = 11cm* |  |  |  |
|  |  |  |  |
| Static contact distance | 4.53 x 10^1^ (p<10^-15^) | 4.81 x 10^-2^ (p<10^-15^) | 40566 |
| Dynamic contact distance | 3.27 x 10^1^ (p<10^-15^) | 1.83 x 10^-1^ (p<10^-15^) | 192457 |
|  |  |  |  |
| *Initial spacing = 14cm* |  |  |  |
|  |  |  |  |
| Static contact distance | 4.63 x 10^1^ (p<10^-15^) | 1.80 x 10^-2^ (p<10^-15^) | 9524 |
| Dynamic contact distance | 3.46 x 10^1^ (p<10^-15^) | 1.22 x 10^-1^ (p<10^-15^) | 102950 |
|  |  |  |  |
| *Initial spacing = 17cm* |  |  |  |
|  |  |  |  |
| Static contact distance | 4.70 x 10^1^ (p<10^-15^) | 5.2 x 10^-3^ (p<10^-4^) | 1421 |
| Dynamic contact distance | 4.08 x 10^1^ (p<10^-15^) | 5.42 x 10^-2^ (p<10^-15^) | 21240 |
|  |  |  |  |

Figure S3 Trends in population model outcomes. Number of infected hosts (grey points), (A-B-C) with a static host contact distance equal to plant spacing and (D-E-F) with dynamic host contact distance. Upper, middle and lower graphs are numbers of infected hosts for distance *x_cc_= 11, 14 and 17cm* and *t> 80, 110 and 140 days* respectively. Green dotted lines show the linear trends obtained from model (s3.2) whereas red lines illustrate the non-linear trends from model (s3.1). (A-B-C) were better exhibited linear trends and were better described by model (s3.2) while (D-E-F) demonstrated non-linear trends and were statistically better described by model (s3.1).
